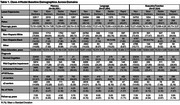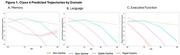# Latent Cognitive Trajectories in Late Life: An Exploratory Analysis

**DOI:** 10.1002/alz70857_105147

**Published:** 2025-12-25

**Authors:** Melisa Lara Gomez, Phoebe Scollard, Sarah Biber, Michael L Cuccaro, Jesse Mez, Seo‐Eun Choi, Brandon Klinedinst, Michael L. Lee, Arthur W. Toga, Aditi Sathe, Michelle J. Clifton, Jane Zyski, Alaina Durant, Shannon Turner, Walter W. Kukull, Andrew J. Saykin, Kwangsik Nho, C. Dirk Keene, Shubhabrata Mukherjee, Eric B Larson, Anja Soldan, Marilyn S. S. Albert, Lori L Beason‐Held, Keenan A. Walker, Luigi Ferrucci, Murat Bilgel, Susan M. Resnick, Yang An, Jason J. Hassenstab, Carlos Cruchaga, Jigyasha Timsina, Corinne D. Engelman, Sterling C Johnson, Richard Mayeux, Katherine A. Gifford, Angela L. Jefferson, Mary Ellen I. Koran, Dandan Liu, Kimberly R. Pechman, Adam Brickman, Annie J. Lee, Jennifer J. Manly, Miguel Arce Renteria, Badri N. Vardarajan, David A. A. Bennett, Julie A Schneider, Lisa L. Barnes, Derek B. Archer, Paul K Crane, Logan Dumitrescu, Timothy J. Hohman, Jo Ellen Wilson

**Affiliations:** ^1^ Vanderbilt Memory & Alzheimer's Center, Vanderbilt University Medical Center, Nashville, TN, USA; ^2^ Department of Medicine, University of Washington School of Medicine, Seattle, WA, USA; ^3^ University of Washington, Seattle, WA, USA; ^4^ Dr. John T. Macdonald Foundation Department of Human Genetics, University of Miami Miller School of Medicine, Miami, FL, USA; ^5^ John P. Hussman Institute for Human Genomics, University of Miami Miller School of Medicine, Miami, FL, USA; ^6^ Department of Neurology, Boston University Chobanian & Avedisian School of Medicin, Boston, MA, USA; ^7^ Laboratory of Neuroimaging, USC Stevens Neuroimaging and Informatics Institute, Keck School of Medicine, University of Southern California, Los Angeles, CA, USA; ^8^ Vanderbilt Memory and Alzheimer's Center, Vanderbilt University School of Medicine, Nashville, TN, USA; ^9^ Vanderbilt Memory and Alzheimer's Center, Vanderbilt University Medical Center, Nashville, TN, USA; ^10^ Vanderbilt University Medical Center, Nashville, TN, USA; ^11^ Department of Epidemiology, School of Public Health, University of Washington, Seattle, WA, USA; ^12^ Department of Medical and Molecular Genetics, School of Medicine, Indiana University, Indianapolis, IN, USA; ^13^ Department of Radiology and Imaging Sciences, Center for Neuroimaging, School of Medicine, Indiana University School of Medicine, Indianapolis, IN, USA; ^14^ Department of Radiology and Imaging Sciences, Indiana Alzheimer's Disease Research Center, Center for Neuroimaging, Indiana University School of Medicine, Indianapolis, IN, USA; ^15^ Center for Computational Biology and Bioinformatics, Indiana University School of Medicine, Indianapolis, IN, USA; ^16^ Department of Pathology, University of Washington, Seattle, WA, USA; ^17^ Department of Medicine, University of Washington, Seattle, WA, USA; ^18^ Department of Neurology, Johns Hopkins University School of Medicine, Baltimore, MD, USA; ^19^ Department of Neurology, The Johns Hopkins University School of Medicine, Baltimore, MD, USA; ^20^ Laboratory of Behavioral Neuroscience, National Institute on Aging Intramural Research Program, National Institutes of Health, Baltimore, MD, USA; ^21^ Laboratory of Behavioral Neuroscience, National Institute on Aging, Intramural Research Program, Baltimore, MD, USA; ^22^ National Institute on Aging Intramural Research Program, National Institutes of Health, Baltimore, MD, USA; ^23^ Department of Neurology, Washington University School of Medicine in St. Louis, St. Louis, MO, USA; ^24^ Washington University School of Medicine, St. Louis, MO, USA; ^25^ Department of Psychiatry, Washington University School of Medicine, St. Louis, MO, USA; ^26^ Department of Population Health Sciences, University of Wisconsin School of Medicine and Public Health, Madison, WI, USA; ^27^ Wisconsin Alzheimer's Disease Research Center, University of Wisconsin School of Medicine and Public Health, Madison, WI, USA; ^28^ Department of Neurology, The New York Presbyterian Hospital, New York, NY, USA; ^29^ Department of Neurology, Taub Institute for Research on Alzheimer's Disease and the Aging Brain, Columbia University, New York, NY, USA; ^30^ Department of Epidemiology, Gertrude H. Sergievsky Center Taub Institute for Research on Alzheimer's Disease and the Aging Brain, New York, NY, USA; ^31^ The Gertrude H. Sergievsky Center, College of Physicians and Surgeons, Columbia University, New York, NY, USA; ^32^ Department of Neurology, The Institute for Genomic Medicine, Columbia University Medical Center and The New York Presbyterian Hospital, New York, NY, USA; ^33^ Department of Neurology, Columbia University Medical Center, New York, NY, USA; ^34^ The Institute for Genomic Medicine, Columbia University Medical Center, New York, NY, USA; ^35^ Department of Neurology, Vanderbilt Memory & Alzheimer's Center, Vanderbilt University Medical Center, Nashville, TN, USA; ^36^ Department of Radiology, Stanford Hospital, Stanford, CA, USA; ^37^ Department of Neurology, Gertrude H. Sergievsky Center, Taub Institute for Research on Alzheimer's Disease and The Aging Brain, Columbia University Medical Center, New York, NY, USA; ^38^ Department of Neurology, Columbia University Medical Center, New York City, NY, USA; ^39^ Department of Neurology, Columbia University, New York, NY, USA; ^40^ Rush Alzheimer's Disease Center, Rush University Medical Center, Chicago, IL, USA; ^41^ Department of Neurology and Pathology, Rush University Medical Center, Chicago, IL, USA; ^42^ Vanderbilt Genetics Institute, Vanderbilt University Medical Center, Nashville, TN, USA; ^43^ Department of General Internal Medicine, University of Washington School of Medicine, Seattle, WA, USA; ^44^ Geriatric Research Education and Clinical Center, Department of Veteran's Affairs, Tennessee Valley Healthcare System, Nashville, TN, USA; ^45^ Center for Cognitive Medicine, Department of Psychiatry and Behavioral Sciences, Vanderbilt University Medical Center, Nashville, TN, USA

## Abstract

**Background:**

Late‐life cognitive trajectories display heterogeneity between individuals and across cognitive domains. We aimed to identify latent classes of cognitive trajectories among three cognitive domains (memory, executive function, and language) and to investigate their association with baseline demographic and clinical characteristics.

**Method:**

Harmonized data were obtained from 14 longitudinal cohorts of cognitive aging and dementia. Participants were restricted to those with at least 3 observations for each domain (memory, language and executive function). We developed three latent class linear mixed models, whereby each composite domain was modeled as both a linear and quadratic function of age. Separate models were developed with 1 through 5 classes. We assessed goodness of fit through a comparison of class proportions, AIC, BIC, and entropy. Baseline variables were compared across classes, with ANOVA for continuous and Chi‐square for categorical variables.

**Result:**

We included 38,358 participants (73% non‐Hispanic white, 41% male, age=73 ± 9 years, education = 15 ± 4, mild cognitive impairment=19%, Alzheimer's disease = 14%, *APOE‐*ε4=31%, *APOE‐*ε2=13%) (Table 1). Across domains, the 4‐class model displayed the best performance (Figure 1) including non‐decliners, slow decliners, steady decliners, and rapid decliners. The memory classes deviated from the other two domains with the rapid decliners class displaying poor performance at younger ages with a less precipitous decline, whereas the rapid decline group in the other two domains declined rapidly with age. Across domains, the non‐decliners had the highest proportion of non‐Hispanic Black, cognitively unimpaired, ε2 carriers, and lowest proportion of ε4 carriers. Slow decliners were older and included more ε2 carriers. Steady decliners had the second highest proportion of AD at baseline and had the highest or second highest proportion of ε4 carriers. Rapid decliners had the highest proportion of AD at baseline, and the highest or second highest proportion of ε4 carriers.

**Conclusion:**

While multiple subgroups move from cognitively normal to dementia, our latent class approach highlights subtypes that decline at different times and at different rates. Future work will seek to integrate disease time into models, clarify the stability of the observed subgroups of decliners, and characterize the neuropathological and neuroimaging features that contribute to the distinct etiologies of decline across domains.